# Human Exonuclease 1 (EXO1) Regulatory Functions in DNA Replication with Putative Roles in Cancer

**DOI:** 10.3390/ijms20010074

**Published:** 2018-12-25

**Authors:** Guido Keijzers, Daniela Bakula, Michael Angelo Petr, Nils Gedsig Kirkelund Madsen, Amanuel Teklu, Garik Mkrtchyan, Brenna Osborne, Morten Scheibye-Knudsen

**Affiliations:** 1 Department of Cellular and Molecular Medicine, Center for Healthy Aging, University of Copenhagen, DK-2200 Copenhagen, Denmark; bakula@sund.ku.dk (D.B.); mapetr@sund.ku.dk (M.A.P.); ngkm@sund.ku.dk (N.G.K.M.); amanuel@sund.ku.dk (A.T.); garik@sund.ku.dk (G.M.); brenna@sund.ku.dk (B.O.); mscheibye@sund.ku.dk (M.S.-K.); 2 Experimental Gerontology Section, Translational Gerontology Branch, National Institute on Aging, NIH, Baltimore, MD 21224, USA

**Keywords:** DNA repair, double strand break repair, exonuclease 1, EXO1, mismatch repair, MMR, NER, nucleotide excision repair, strand displacements, TLS, translesion DNA synthesis

## Abstract

Human exonuclease 1 (EXO1), a 5′→3′ exonuclease, contributes to the regulation of the cell cycle checkpoints, replication fork maintenance, and post replicative DNA repair pathways. These processes are required for the resolution of stalled or blocked DNA replication that can lead to replication stress and potential collapse of the replication fork. Failure to restart the DNA replication process can result in double-strand breaks, cell-cycle arrest, cell death, or cellular transformation. In this review, we summarize the involvement of EXO1 in the replication, DNA repair pathways, cell cycle checkpoints, and the link between EXO1 and cancer.

## 1. Introduction

**Figure 1:**
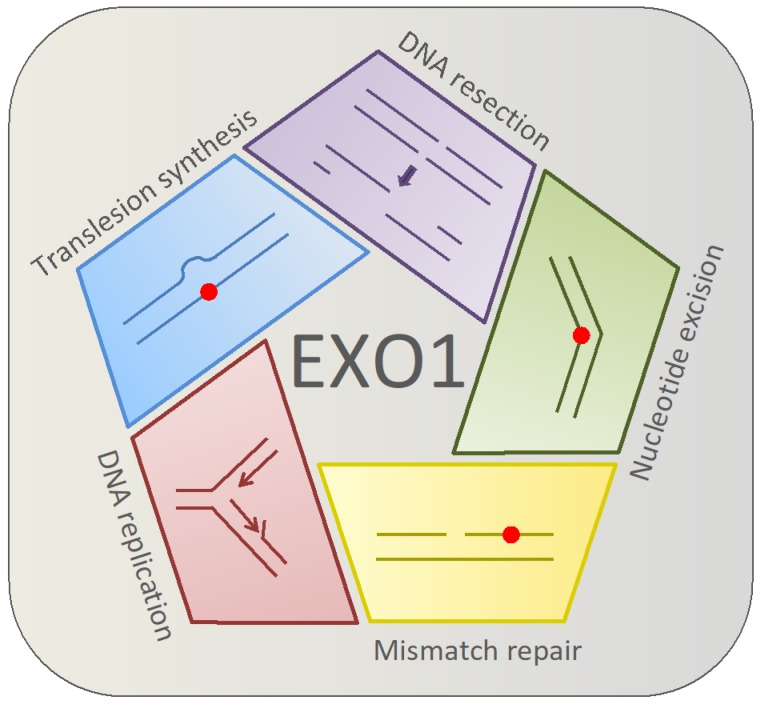
Human EXO1 participates in both replicative and post-replicative processes. In the replicative process, EXO1 contributes to DNA replication by assisting in the removal of mismatches, bypassing the lesion using translesion synthesis, or by assisting with nucleotide excision repair by activating the NER repair pathway. EXO1 also has a role in DNA resection during the process of homologous recombination.

Human exonuclease 1 (EXO1) contributes to checkpoint progression and to several DNA repair pathways involved in reducing DNA replication stress, for example, in mismatch repair (MMR), translesion DNA synthesis (TLS), nucleotide excision repair (NER), double-strand break repair (DSBR), and checkpoint activation to restart stalled DNA forks [1,2,3,4,5,6]. The multifarious and crucial roles of EXO1 in these DNA repair pathways are summarized in Figure 1.

**Figure 2:**
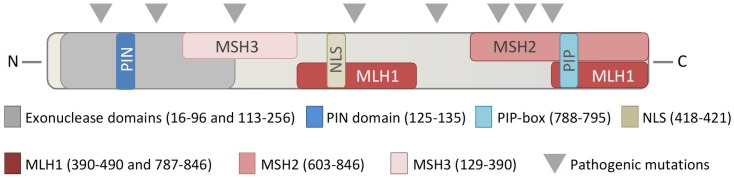
Interaction domains in EXO1. Schematic overview of the relevant interaction domains in the human EXO1 protein, denoting interaction domains with mismatch repair proteins MSH3, MLH1, MSH2, and other significant interaction regions, including with PARP1, PCNA, and the nuclear localization signal (NLS).

EXO1 is a member of the Rad2/XPG family of nucleases [7], and contains an active domain, located at the *N*-terminal region of the protein (Figure 2). The EXO1 transcript has 5′→3′ exonuclease activity, as well as 5′ structure specific DNA endonuclease activity and 5′→3′ RNase H activity [7,8]. EXO1 has a high affinity for processing double stranded DNA (dsDNA), DNA nicks, gaps, and DNA fork structures, and is involved in resolving double Holliday junctions [9,10,11,12]. During DNA replication in the S-phase of the cell cycle, a polymerase can incorporate a mismatched DNA base or encounter secondary DNA structures, which can stall the replication fork and lead to replication stress. The collapse of a replication fork can have severe consequences, and failure to restart a stalled fork may lead to double-strand breaks, chromosomal rearrangement, cell-cycle arrest, cell death, or malignant transformation [13,14]. 

The contribution of EXO1 in the safeguarding stability of the genome during DNA replicative and post-replicative processes is well-established. EXO1 activity contributes to several DNA repair processes; however, it is not clear if the absence or malfunction of EXO1 can contribute to cancer development. We will herein examine the putative wider roles of EXO1 as a guardian of our genome and investigate its possible role in cancer progression and initiation. 

## 2. DNA Replication

Enzymes able to metabolize DNA are required for modulating DNA replication. EXO1 is intricately involved in this process both as an enzyme involved in replication and in DNA repair pathways such as homologous recombination, but it is also an essential enzyme in the replication process, such as DNA strand displacement. Strand displacement describes the removal of single stranded RNA or DNA from an RNA:DNA or DNA:DNA duplex, a process required for multiple essential cellular processes, such as DNA replication and DNA repair. Accordingly, flap structure-specific endonuclease 1 (FEN1), EXO1, and polymerase δ are the main factors in primer removal and Okazaki fragment maturation at the lagging strand in the process of strand displacement during replication [8,15,16,17,18,19]. In yeast, EXO1 can substitute for RAD27 (FEN1 is the human homolog) in RNA primer removal [11]. Indeed, in vitro assays suggest that 5’ flaps (<5 nt) generated by polymerase δ during replication are efficiently removed by FEN1 or EXO1 [9,11,15,16]. The 3’-exonuclease activity of polymerase δ avoids excessive strand displacement [19]. Deletion of POL32 (third subunit of polymerase δ) can suppress the lethality of growth defects of RAD27 and polymerase δ D520V mutants in yeast (defective for RAD27 and the 3’→5’ exonuclease of polymerase δ) [20]. In support of this observation, synthetic lethality is seen in yeast *exo1Δ*, *rad27Δ* double knockout cells [17,18,19,21]. This suggests significant overlap in the functionality of these enzymes. Accordingly, both human FEN1 and EXO1 have weak flap activity at long 5’ flap overhangs (5–20 nucleotides), but efficiently remove mono- or dinucleotide overhangs [8,11,15]. Further, both EXO1 and FEN1 have been demonstrated to have RNA and DNA displacement activity in vitro [8,11,15,21]. In addition, in biochemical assays, it was demonstrated that the human RecQL helicases, RECQ1 and WRN, physically and functionally interact with human EXO1 and increase its exo- and endonucleolytic incision activities catalyzed by EXO1 [22,23]. Both RecQL helicases efficiently unwind the 5’ flap DNA substrate [22,23], which is a critical intermediate that arises during the DNA strand displacement process. Therefore, the combined helicase and physical interaction of EXO1 with RECQL1 or WRN may play an important role in the enhancement of DNA strand displacement, such as that occurring during lagging strand DNA synthesis at the replication fork, or during the DNA repair (for example, long patch base excision repair) that also potentially leads to strand displacement. These findings highlight the role of EXO1 in DNA replication and underscore the need for a multitude of enzymatic processes required for human DNA synthesis. Longer DNA flaps with more than 25 nucleotides are processed in the presence of RPA, FEN1, and helicase partner with either the ATP-dependent helicase Petite Integration Frequency 1 (PIF1) or DNA replication helicase/nuclease 2 (DNA2) in vitro [24,25,26,27,28]. However, it was recently demonstrated that DNA2 and RPA can process long flaps independent of RAD27 in yeast [29,30]. In vitro data suggest that POL32 has no effect on the generation of short flaps. Notably, longer flaps only accumulate in the presence of POL32, indicating that polymerase δ and FEN1 team up in short flap removal. The role of EXO1 in the removal of long DNA flaps of more than 25 nucleotides has not yet been extensively studied [9,11]. It is possible that EXO1 could potentially act as a back-up to FEN1 during circumstances of cellular stress. However, it has to be taken into account that the actual contribution of EXO1 in humans remains understudied and there is much scope for further work in this area.

## 3. Mismatch Repair 

High-fidelity DNA replication is required to maintain an unaltered genetic code during cell division. The MMR pathway is a post-replicative DNA repair system, which mainly corrects DNA polymerase slippage and damaged bases, such as chemically-induced base adducts; base mismatches; and base insertions, deletions, and loops. The MMR pathway consists of several steps, which are detailed below. The initial recognition step of eukaryotic MMR utilizes the MutSα complex made up of mutS homolog 2 (MSH2) and mutS homolog 6 (MSH6) or MutSβ complex (MSH2 and mutS homolog 3 (MSH3)). The MutSα mainly recognizes single base mismatches, while the MutSβ complex detects larger lesions, insertion/deletions, or loops [31,32,33]. The MutSα or β complex operates by binding to the DNA mismatched base or DNA distortion. Following the initial DNA distortion recognition, the MutLα complex (heterodimer of MLH1/Postmeiotic Segregation Increased 2 (PMS2)), proliferating cell nuclear antigen (PCNA), and replication factor C (RFC) are recruited. MutSα or Mutsβ forms a tetrameric complex with MutLα at the site of the replication error. In the presence of PCNA and RFC, the MutLα nicks the DNA at 3’ or 5’ to the lesion by use of the intrinsic endonuclease activity in PMS2. EXO1’s contribution to the MMR was identified in fission yeast (*Schizosaccharomyces pombe*) after it was co-purified with mismatch repair factor MSH2 [2]. EXO1 is the only known nuclease active in the MMR pathway by interacting with the mismatch repair factors mutL homolog 1 (MLH1), MSH2, MSH3, and PCNA (Table 1) [34,35,36,37,38,39,40,41,42]. EXO1 is recruited to excise the newly synthesized DNA containing the replication error in a MutSα or β, and in a MutLα-dependent manner. Additional factors, such as replication protein A (RPA), guide the resection of the single stranded DNA (ssDNA) intermediates during the DNA repair process to avoid the formation of secondary DNA structures or excessive DNA degradation [43]. The repair reaction is completed by the joint activities of the PCNA and DNA polymerase δ/ε resynthesizing the DNA, and DNA ligase I sealing the nick [44]. Malfunction of MMR is associated with increased microsatellite instability (MSI), a hallmark of certain types of colon cancer, such as hereditary nonpolyposis colorectal cancer (HNPCC), also known as Lynch Syndrome (Online Mendelian Inheritance in Man (OMIM) #120435) [45,46,47]. 

More recently, it was shown that MMR occurs in the absence of EXO1 [48,49], suggesting that a proportion of MMR is EXO1-independent and relies on either strand displacement or involvement of other helicases or nucleases. Indeed, several members of the RecQL family of helicases have been proposed to be involved in MMR. The WRN helicase/exonuclease interacts with MutL1α, MutSα, MutSβ, and RPA. However, only MutSα, MutSβ, and RPA stimulate the DNA helicase activity of WRN on naked DNA [50,51,52]. Interestingly, it is reported that in some cases, cells from patients with Werner Syndrome (OMIM#277700) show a malfunction in the MMR [32,53,54,55]. Nonsense mutations in the BLM gene lead to Bloom Syndrome disease (OMIM#210900). Some Bloom Syndrome cases show immunodeficiency and increased MSI [56]. Furthermore, the RECQL helicases, RECQL1 and BLM, physically interact with MutLα, MutSα, and RPA [23,57,58,59,60]. Only MutSα and RPA enhance the helicase activity of RECQ1 and BLM [23,58,59,60,61]. However, the above is in contrast to in vitro assays with human cell extracts of BLM^−/−^ and WRN^−/−^ that show no defective MMR [62,63]. Altogether, this suggests that the RECQL helicase has some stimulatory role in the MMR pathway, but does not have a significant contribution in the absence of EXO1. Nonetheless, deficiency in the MMR pathway in human cell lines in the absence of helicases WRN or BLM in combination with the depletion of EXO1 has not been reported. In addition, some nucleases have been suggested to back up MMR in the absence of EXO1, including the MRE11 homolog A (MRE11) and FAN1 (FANCD2/FANCI-Associated Nuclease 1) [64]. The contribution of MRE11 to the MMR pathway and to MSI has recently been reviewed [32]. A recent study showed that overexpression of the human polymerase δ D316A;E318A mutant resulted in mild MMR deficiency [65]. In vitro experiments with cell extracts show that the overexpression or addition of human EXO1 protein compliments the mild mutator phenotype of polymerase δ D316A;E318A, indicating that EXO1 can provide backup to polymerase δ in its MMR activity [65]. It has been suggested that the polymersase δ strand displacement activity may indeed depend on the endo-nuclease activity of MutLα in the absence of EXO1 [66]; however, the mechanism is so far unknown. While the role of EXO1 in MMR is well-established, EXO1-independent MMR in eukaryotic cells is still not understood. 

## 4. Translesion DNA Synthesis

Translesion DNA synthesis (TLS) describes the process by which a DNA polymerase can synthesize a DNA strand across a lesion on the template strand. This process is critical to maintaining functional DNA replication in the face of genotoxic stress and may act as a pathway to cope with ultra violet (UV) induced DNA damage [3]. Indeed, in human cell lines, it was demonstrated that EXO1 recruits the TLS DNA polymerases κ and ι to sites of UV damage [3]. Interestingly, an inactivating mutation in the aspartate at position 173 to alanine in EXO1 (EXO1-D173A) results in an inability to recruit the TLS polymerase κ/ι to the damage site, suggesting an active role of EXO1 in TLS [3]. Notably, in yeast, the EXO1 mutant strain (FF447AA) shows defective MMR due to the loss of interaction with MLH1, but is still active in TLS [67]. However, it remains unclear if such an EXO1 variant can assist in UV-induced TLS in mammals. In addition, the yeast 9-1-1 complex (three distinct subunits complex of Ddc1, Mec3, and Rad17 in yeast and RAD9, HUS1, and RAD1 in humans) and EXO1 also contribute to an error-free TLS pathway in a PCNA monoubiquitinylation manner that makes use of undamaged sister chromatids as templates for repair [68]. Overall, EXO1 appears to have an emerging role in TLS, requiring further investigation.

## 5. Nucleotide Excision Repair 

UV radiation from sunlight mainly damages DNA by causing cyclobutane pyrimidine dimers, and 6–4 photoproducts, lesions typically repaired by the nucleotide excision repair pathway (NER) independent of replication [69]. However, during the S-phase of the cell cycle, UV radiation-induced base lesions block DNA replication. EXO1 belongs to the same family of nucleases as xeroderma pigmentosum complementation group G (XPG), a protein involved in NER. Accordingly, cells damaged by UV exposure and inhibited in translesion synthesis show an accumulation of EXO1 at the DNA damage sites [3]. Indeed, an additive UV-sensitivity effect is observed in yeast when both *rad2* (XPG homolog in human) and *exo1* are knocked out [69]. In addition, yeast EXO1 competes with the translesion synthesis pathways, and converts the NER intermediates to long ssDNA gaps, leading to checkpoint activation [4]. In human cell lines, EXO1 enlarges ssDNA gaps to stretch over 30 nucleotides long to activate the ATR checkpoint [70]. The contribution of EXO1 to NER is likely limited to enlarging the DNA gaps that occur as part of NER leading to checkpoint activation; although this is not well-understood.

## 6. Homologous Recombination and DNA End Resection 

Homologous recombination (HR) is an essential process involved in the repair of double strand DNA breaks, mainly in the S and G2-phases of the cell cycle. A possible piece of evidence suggesting the involvement of EXO1 in double strand DNA repair is the observation that *Exo1^null/null^* mice show an increase in chromosomal breaks and base substitution, and predominately develop lymphomas [71]. In addition, human cell lines depleted in EXO1 exert chromosomal instability and demonstrate a hypersensitivity to ionizing radiation (IR), a hallmark of cells defective in homologous recombination [5]. This provides support that EXO1 is required for the HR repair of DSBs in human cells. In contrast, yeast *exo1^-/-^* has no significant defect in recombinational repair, with only minor defects in DNA end processing [16,18,19,72]. Data also suggests that EXO1 is involved in DNA damage signaling upon replication fork stalling [73]. The 5′→3′ DNA resection of DSB ends to produce a 3’ single stranded DNA overhang is a critical step in the repair of DSBs by HR [74]. In mouse embryonic fibroblasts (MEF), *Exo1^null/null^* cells showed a defect in the DNA damage response [71]. Treatment of *Exo1^null/null^* cells with the topoisomerase inhibitor camptothecin, which creates single strand breaks (SSB) that ultimately lead to DSB during the S-phase, results in a reduction in phosphorylated RPA (pRPA) foci at the DSBs [71]. Recruitment of pRPA is regulated by DNA damage response protein-kinases, such as ataxia telangiectasia mutated (ATM) and ataxia telangiectasia mutated and Rad3 related (ATR) [71]. PARP1, a factor involved in DSB repair, physically interacts with EXO1 at the PAR interaction motif (PIN) at the N-terminus of EXO1 [75,76] and stimulates EXO1 in its 5’ excision activity in an in vitro MMR assay [77]. Poly (ADP-Ribose) Polymerase 1 (PARP1) promotes PAR-mediated polyADP-ribosylation (PARylation) recruitment to the DNA damage site, followed by additional DNA repair factors [75,76]. The EXO1-R93G variant, mutated in its PIN domain, is poorly recruited to damaged DNA [76]. This suggests that PARP1 is potentially essential in the early recruitment of EXO1. The interplay between the MRE11-RAD50-NBS1 (MNR)-complex and EXO1 is well-documented [78,79,80,81] and deletion in *Mre11*, *Rad50*, or *Nbs1* genes has been shown to be lethal in mice [82]. Mice that carry a hypomorphic allele of *Nbs1* (*Nbs1^ΔB/ΔB^*) are viable, but show severe developmental impairment, embryonic death, and chromosomal instability when *Exo1* is lost [82]. The *Nbs1^ΔB/ΔB^* MEFs depleted in EXO1 strongly influenced DNA replication, DNA repair, checkpoint signaling, and the DNA damage response [82]. 

The single-stranded DNA binding protein RPA has a central role in DNA replication, DNA repair, recombination, and DNA resection [83]. DNA resection after double strand DNA breaks is proposed to occur via two different routes. In the RPA-BLM-DNA2-MRN mediated route, RPA stimulates DNA unwinding by the DNA helicase BLM in a 5’→3’ direction, leading to the formation of single stranded DNA that can be resected by the nuclease DNA2 [79]. The other resection route is mediated by EXO1 and is stimulated by BLM, MRN, and RPA [79]. Indeed, yeast depleted in RPA and loss of *Mre11* eliminates both SGS1-DNA2 mediated and EXO1-dependent resection pathways [43], suggesting that RPA and MRN are essential for resection. DNA-resection by EXO1 is probably inhibited by the DNA binders RPA, Ku70/80, and/or *C*-terminal-binding protein interacting protein (CtIP) (the yeast homolog is SAE2) [43,81,84,85,86]. Accordingly, in nonhomologous end joining, the Ku70/80 heterodimer protects the DNA in a complex with DNA-PKcs for DNA end resection [86,87,88]. Therefore, EXO1 has a limited role in this pathway. In contrast, EXO1 likely collaborates in an alternative end joining pathway with the WRN in trimming the DNA ends [89,90,91]. EXO1 interacts with WRN and enhances the exonuclease activity of EXO1 by the *C*-terminal region of WRN. Biochemical assays suggest that WRN and EXO1 function in replication stress, where WRN enhances EXO1 in processing stalled forks or regressed replication forks [92]. More recently, it was shown that the WRN exonuclease activity prevents unscheduled degradation by MRE11 and EXO1 during replication re-start [93]. Human cells depleted in WRN show an enhanced degradation of the nascent DNA strand by MRE11 and EXO1 after camptothecin treatment [93]. In summary, EXO1 is required for homologous recombination, while it is less essential for nonhomologous end joining.

## 7. Cell Cycle Regulation

Several lines of evidence suggest that EXO1 may be a central regulator of the cell cycle. For example, in S-phase, EXO1 co-localizes with MMR protein MSH2 and cell cycle regulator PCNA [39]. In humans, EXO1 interacts physically with PCNA via the PCNA-interacting protein (PIP box) motif located in the C-terminal region of EXO1 [40,41,94]. Indeed, PCNA stimulates the exonuclease activity of EXO1 on dsDNA substrates [95]. 

Further evidence for a regulatory function of EXO1 in the cell cycle comes from yeast, where the absence of cell cycle regulator 14-3-3 leads to checkpoint defects [96]. In humans, EXO1 physically interacts with six of the seven 14-3-3 isoforms and is stimulated by isoform 14-3-3η and 14-3-3σ in its exonuclease activity in vitro [96]. The EXO1-dependent resection pathway is restrained by 14-3-3σ, thereby counteracting EXO1 stimulation by PCNA [97,98]. In addition to the 14-3-3 complex, the 9-1-1 complex functions on the crossroads between checkpoint activation and DNA repair, and stimulates DNA resection of yeast EXO1 [99,100]. In total, EXO1 physically and functionally interacts with multiple central proteins involved in cell-cycle regulation and is therefore likely to be important in these processes.

## 8. Link to Cancer

EXO1 has been associated with different types of cancers, including Lynch Syndrome, breast, ovarian, lung, pancreatic, and gastric tract cancer (see Table 2) [101,102,103,104,105,106,107,108,109,110,111,112,113,114,115,116,117]. Lynch Syndrome is commonly caused by mutations in the MLH1 and MSH2 genes in humans that give rise to almost two-thirds of all Lynch Syndrome cases [45,118]. A hallmark of MMR deficiency in MSH2^-/-^ and MLH1^-/-^ cells is the presence of MSI, leading to increased chromosomal instability, which is believed to be the underlying molecular driver of tumor formation in Lynch syndrome [21,45,118]. Several studies have been conducted on single-nucleotide polymorphisms (SNP) in EXO1 related to MSI in tumors in humans; however, it remains inconclusive if EXO1 defects contribute to MSI. However, in genomic-wide association studies (GWAS), specific mutations in EXO1 have been identified as risk alleles for the development of multiple types of cancer [112,116]. Notably, at least some of these mutations can lead to the loss of protein function. For example, the A153V and N279S mutations are located in the active nuclease domain (as highlighted in both Table 2, and shown graphically in Figure 2) and are likely related to the malfunction of the nuclease activity of EXO1. Other mutations in EXO1, including T439M, E670G, and P757L, are located in the MLH1 and MSH2 binding domains (Figure 2). One of the most studied mutations is the E109K, which was suggested to be dysfunctional in the nuclease domain [71,101]. However, biochemistry studies revealed that EXO1 E109K is functional in its nuclease activity [119,120]. The mutation is located in the EXO1 PAR-binding motif, and therefore potentially not recruited to sites of DNA damage [76]. The clinical data is supported by mouse models, where the loss of *Exo1* leads to an increased incidence of lymphomas, but interestingly not to increased MSI [71]. Pathogenic mutations in both introns, exons and the untranslated regions of EXO1 have been described [112]. Nevertheless, the overexpression of EXO1 has also been reported in several other cancers, which in part is related to increased DNA repair activity [121,122,123,124]. However, EXO1 is in general expressed at low levels, independent of the cell-cycle progression or proliferative status of the cell, and increased levels of EXO1 are harmful and lead to genomic instability [6]. Several other nucleases including FEN1 and MRE11 have also been demonstrated to have elevated levels of expression in tumors [125,126,127]. Clearly, the connection between EXO1 and cancer has been established and could represent a druggable target in cancers where the EXO1 protein is overexpressed.

## 9. Conclusions and Perspectives

Evidently, EXO1 is a central player in DNA metabolic processes. As elucidated herein, EXO1 contributes to several DNA repair pathways, which safeguard DNA replication, including MMR, TLS, HR, and cell cycle regulation (Figure 2). Replication fork collapse and checkpoint failure during DNA replication can lead to chromosomal instability or abnormal DNA repair, leading to translocation, transformation, and cell death, all processes where EXO1 has been implicated. 

Nonetheless, several questions remain to be answered. For example, given the putative central role of EXO1, it remains a mystery why the knockout of EXO1 in mice, as well as loss of function, leads to a relatively mild phenotype. Further, the mechanism of EXO1-independent MMR is still unclear, particularly regarding at what point this specific pathway is active. Given the biochemical activity of EXO1, it is possible that an unknown helicase or exonuclease can contribute to MMR repair in the absence of EXO1. DNA polymerase δ is a strong candidate, as well as the helicases BLM and/or WRN with minor contributions [50,51,52,53,54,56,57,58,65]. However, unknown contributors with a more prominent role in MMR may still remain to be discovered. 

EXO1 gene variants have been associated with different types of cancers. Interestingly, large GWAS analyses support that specific mutations in domains required for interaction with other proteins in EXO1 are more commonly occurring in particular types of cancer, as summarized in Table 2 [107,108,109,110,111,112,116]. The central role of EXO1 in replication and post-replication processes, including checkpoint activation, suggests that EXO1 dysfunction could alter other DNA repair pathways, leading to replication stress followed by genomic instability and the development of cancer. Deregulation of EXO1 protein levels in tumors is commonly reported [121,122]. Furthermore, EXO1 has been addressed as a candidate gene in cancer therapeutics through its increased expression in tumors [123]. Given the large number of processes that involve EXO1, it is not surprising that EXO1 has emerged as a critical protein in cancer research. Nevertheless, several enigmas remain and the EXO1 field is fertile for future explorations.

## Figures and Tables

**Table 1:** EXO1 interactor proteins in humans and yeast. Significant interaction partners of EXO1 in humans and yeast during different cellular processes.

Repair Process	EXO1 Interaction Proteins in Human	Reference	EXO1 Interaction Proteins in Yeast	Reference
Mismatch repair	MSH2 MSH3 MLH1 PCNA	[36,38] [32,33] [38,41] [40]	MSH2 MSH3 MLH1	[2,34] [72] [72]
Homologous recombination /DNA replication/DNA end resection	PARP1 BLM WRN RECQ1 CTIP	[75,76] [57,79] [22] [23] [85]	SGS1 SAE2	[74] [74]
Cell cycle regulation	PCNA 14-3-3η 14-3-3σ	[40,41,95] [97,98] [97,98]	9-1-1 14-3-3	[99,100] [96]

**Table 2:** Mutations in EXO1 in relation to different cancers. Represents the most commonly reported point mutations in EXO1 in relation to different cancer types. Abbreviations: CRC- colorectal cancer, IC- cancer of the small intestine, BC- breast cancer, PC- pancreatic cancer, GC- gastric cancer, LC- lung cancer, HCC- hepatocellular carcinoma, OC- oral cancer, and CC- cervical cancer.

Mutations in EXO1 Region	Corresponding DNA Sequence Mutation	Reported SNP	Coding and Non-Coding Region	Type of Cancer/Remark	Reference
p.E109K	c.326A>G	rs756251971	exon	CRC	[101]
p.A153V	c.458C>G	rs143955774	exon	CRC, IC Combined with pol**ε** c.1373A>T, p.Y458F	[102]
p.N279S	c.836A>G	rs4149909	exon	BC, PC	[103,104]
p.T439M	c.1317G>A	rs4149963	exon	CRC	[105]
p.E589K	c.1765G>A	rs1047840	exon	GC, LC, HCC, Melanoma, Glioblastoma	[106,107,108,109,110,111,112,113]
p.E670G	c.2009A>G	rs1776148	exon	GC, BC, OC, LC, Melanoma, Glioblastoma	[106,107,108,109,111,112,113]
p.R723G/p.R723S	c.2167C>A/c.2167C>T	rs1635498	exon	GC, BC, OC, LC	[107,108,109,111,112]
p.P757L	c.2270C>T	rs9350	exon	CRC, PC, GC, OC, LC, BC, Melanoma	[105,107,108,109,111,112,113,114]
Non coding region	c.2212-1G>C	rs4150000	Intron, splicing variant	PC	[115]
		rs72755295	Intron, splicing variant		[116]
		rs1776177	UTR region	GC, BC, OC, LC	[107,108,109,111]
		rs1635517	UTR region	GC, BC, OC, LC	[107,108,109,111]
		rs3754093	UTR region	GC, BC, OC, LC	[107,108,109,111]
		rs851797	UTR region	GC, BC, OC, LC	[107,108,109,111,112,117]
	c.C-908G	rs10802996	UTR region	CC, GC, BC, OC, LC	[107,108,109,111,112]

## References

[1] Cotta-Ramusino C., Fachinetti D., Lucca C., Doksani Y., Lopes M., Sogo J., Foiani M. (2005). Exo1 processes stalled replication forks and counteracts fork reversal in checkpoint-defective cells. Mol. Cell.

[2] Szankasi P., Smith G.R. (1995). A role for exonuclease I. from S. pombe in mutation avoidance and mismatch correction. Science.

[3] Sertic S., Mollica A., Campus I., Roma S., Tumini E., Aguilera A., Muzi-Falconi M. (2018). Coordinated Activity of Y Family TLS Polymerases and EXO1 Protects Non-S Phase Cells from UV-Induced Cytotoxic Lesions. Mol. Cell.

[4] Qiu J., Guan M.X., Bailis A.M., Shen B. (1998). Saccharomyces cerevisiae exonuclease-1 plays a role in UV resistance that is distinct from nucleotide excision repair. Nucleic Acids Res..

[5] Bolderson E., Tomimatsu N., Richard D.J., Boucher D., Kumar R., Pandita T.K., Burma S., Khanna K.K. (2010). Phosphorylation of Exo1 modulates homologous recombination repair of DNA double-strand breaks. Nucleic Acids Res..

[6] Keijzers G., Liu D., Rasmussen L.J. (2016). Exonuclease 1 and its versatile roles in DNA repair. Crit. Rev. Biochem. Mol. Biol..

[7] Wilson D.M., Carney J.P., Coleman M.A., Adamson A.W., Christensen M., Lamerdin J.E. (1998). Hex1: A new human Rad2 nuclease family member with homology to yeast exonuclease 1. Nucleic Acids Res..

[8] Qiu J., Qian Y., Chen V., Guan M.X., Shen B. (1999). Human exonuclease 1 functionally complements its yeast homologues in DNA recombination, RNA primer removal, and mutation avoidance. J. Biol. Chem..

[9] Lee B.I., Wilson D.M. (1999). The RAD2 domain of human exonuclease 1 exhibits 5’ to 3’ exonuclease and flap structure-specific endonuclease activities. J. Biol. Chem..

[10] Genschel J., Modrich P. (2003). Mechanism of 5’-directed excision in human mismatch repair. Mol. Cell.

[11] Keijzers G., Bohr V.A., Rasmussen L.J. (2015). Human exonuclease 1 (EXO1) activity characterization and its function on flap structures. Biosci. Rep..

[12] Zakharyevich K., Ma Y., Tang S., Hwang P.Y., Boiteux S., Hunter N. (2010). Temporally and biochemically distinct activities of Exo1 during meiosis: Double-strand break resection and resolution of double Holliday junctions. Mol. Cell.

[13] Segurado M., Diffley J.F. (2008). Separate roles for the DNA damage checkpoint protein kinases in stabilizing DNA replication forks. Genes Dev..

[14] Kim H.S., Nickoloff J.A., Wu Y., Williamson E.A., Sidhu G.S., Reinert B.L., Jaiswal A.S., Srinivasan G., Patel B., Kong K. (2017). Endonuclease EEPD1 Is a Gatekeeper for Repair of Stressed Replication Forks. J. Biol. Chem..

[15] Rossi M.L., Bambara R.A. (2006). Reconstituted Okazaki fragment processing indicates two pathways of primer removal. J. Biol. Chem..

[16] Moreau S., Morgan E.A., Symington L.S. (2001). Overlapping functions of the Saccharomyces cerevisiae Mre11, Exo1 and Rad27 nucleases in DNA metabolism. Genetics.

[17] Sparks J.L., Chon H., Cerritelli S.M., Kunkel T.A., Johansson E., Crouch R.J., Burgers P.M. (2012). RNase H2-initiated ribonucleotide excision repair. Mol. Cell.

[18] Fiorentini P., Huang K.N., Tishkoff D.X., Kolodner R.D., Symington L.S. (1997). Exonuclease I of Saccharomyces cerevisiae functions in mitotic recombination in vivo and in vitro. Mol. Cell. Biol..

[19] Llorente B., Symington L.S. (2004). The Mre11 nuclease is not required for 5’ to 3’ resection at multiple HO-induced double-strand breaks. Mol. Cell. Biol..

[20] Stith C.M., Sterling J., Resnick M.A., Gordenin D.A., Burgers P.M. (2008). Flexibility of eukaryotic Okazaki fragment maturation through regulated strand displacement synthesis. J. Biol. Chem..

[21] Tishkoff D.X., Filosi N., Gaida G.M., Kolodner R.D. (1997). A novel mutation avoidance mechanism dependent on S. cerevisiae RAD27 is distinct from DNA mismatch repair. Cell.

[22] Sharma S., Sommers J.A., Driscoll H.C., Uzdilla L., Wilson T.M., Brosh R.M. (2003). The exonucleolytic and endonucleolytic cleavage activities of human exonuclease 1 are stimulated by an interaction with the carboxyl-terminal region of the Werner syndrome protein. J. Biol. Chem..

[23] Doherty K.M., Sharma S., Uzdilla L.A., Wilson T.M., Cui S., Vindigni A., Brosh R.M. (2005). RECQ1 helicase interacts with human mismatch repair factors that regulate genetic recombination. J. Biol. Chem..

[24] Pike J.E., Henry R.A., Burgers P.M., Campbell J.L., Bambara R.A. (2010). An alternative pathway for Okazaki fragment processing: Resolution of fold-back flaps by Pif1 helicase. J. Biol. Chem..

[25] Rossi M.L., Pike J.E., Wang W., Burgers P.M., Campbell J.L., Bambara R.A. (2008). Pif1 helicase directs eukaryotic Okazaki fragments toward the two-nuclease cleavage pathway for primer removal. J. Biol. Chem..

[26] Gloor J.W., Balakrishnan L., Campbell J.L., Bambara R.A. (2012). Biochemical analyses indicate that binding and cleavage specificities define the ordered processing of human Okazaki fragments by Dna2 and FEN1. Nucleic Acids Res..

[27] Munashingha P.R., Lee C.H., Kang Y.H., Shin Y.K., Nguyen T.A., Seo Y.S. (2012). The trans-autostimulatory activity of Rad27 suppresses dna2 defects in Okazaki fragment processing. J. Biol. Chem..

[28] Zaher M.S., Rashid F., Song B., Joudeh L.I., Sobhy M.A., Tehseen M., Hingorani M.M., Hamdan S.M. (2018). Missed cleavage opportunities by FEN1 lead to Okazaki fragment maturation via the long-flap pathway. Nucleic Acids Res..

[29] Duxin J.P., Moore H.R., Sidorova J., Karanja K., Honaker Y., Dao B., Piwnica-Worms H., Campbell J.L., Monnat R.J., Stewart S.A. (2012). Okazaki fragment processing-independent role for human Dna2 enzyme during DNA replication. J. Biol. Chem..

[30] Levikova M., Cejka P. (2015). The Saccharomyces cerevisiae Dna2 can function as a sole nuclease in the processing of Okazaki fragments in DNA replication. Nucleic Acids Res..

[31] Hsieh P., Yamane K. (2008). DNA mismatch repair: Molecular mechanism, cancer, and ageing. Mech. Ageing Dev..

[32] Liu D., Keijzers G., Rasmussen L.J. (2017). DNA mismatch repair and its many roles in eukaryotic cells. Mutat. Res..

[33] Kunkel T.A., Erie D.A. (2005). DNA mismatch repair. Annu. Rev. Biochem..

[34] Tishkoff D.X., Boerger A.L., Bertrand P., Filosi N., Gaida G.M., Kane M.F., Kolodner R.D. (1997). Identification and characterization of Saccharomyces cerevisiae EXO1, a gene encoding an exonuclease that interacts with MSH2. Proc. Natl. Acad. Sci. USA.

[35] Tran H.T., Gordenin D.A., Resnick M.A. (1999). The 3’-->5’ exonucleases of DNA polymerases delta and epsilon and the 5’-->3’ exonuclease Exo1 have major roles in postreplication mutation avoidance in Saccharomyces cerevisiae. Mol. Cell. Biol..

[36] Schmutte C., Sadoff M.M., Shim K.S., Acharya S., Fishel R. (2001). The interaction of DNA mismatch repair proteins with human exonuclease I. J. Biol. Chem..

[37] Schmutte C., Marinescu R.C., Sadoff M.M., Guerrette S., Overhauser J., Fishel R. (1998). Human exonuclease I interacts with the mismatch repair protein hMSH2. Cancer Res..

[38] Jäger A.C., Rasmussen M., Bisgaard H.C., Singh K.K., Nielsen F.C., Rasmussen L.J. (2001). HNPCC mutations in the human DNA mismatch repair gene hMLH1 influence assembly of hMutLalpha and hMLH1-hEXO1 complexes. Oncogene.

[39] Amin N.S., Nguyen M.N., Oh S., Kolodner R.D. (2001). exo1-Dependent mutator mutations: Model system for studying functional interactions in mismatch repair. Mol. Cell. Biol..

[40] Nielsen F.C., Jäger A.C., Lützen A., Bundgaard J.R., Rasmussen L.J. (2004). Characterization of human exonuclease 1 in complex with mismatch repair proteins, subcellular localization and association with PCNA. Oncogene.

[41] Liberti S.E., Andersen S.D., Wang J., May A., Miron S., Perderiset M., Keijzers G., Nielsen F.C., Charbonnier J.B., Bohr V.A. (2011). Bi-directional routing of DNA mismatch repair protein human exonuclease 1 to replication foci and DNA double strand breaks. DNA Repair.

[42] Goellner E.M., Putnam C.D., Graham W.J., Rahal C.M., Li B.Z., Kolodner R.D. (2018). Identification of Exo1-Msh2 interaction motifs in DNA mismatch repair and new Msh2-binding partners. Nat. Struct. Mol. Biol..

[43] Chen H., Lisby M., Symington L.S. (2013). RPA coordinates DNA end resection and prevents formation of DNA hairpins. Mol. Cell.

[44] Li G.M. (2008). Mechanisms and functions of DNA mismatch repair. Cell Res..

[45] Pedroni M., Tamassia M.G., Percesepe A., Roncucci L., Benatti P., Lanza G., Gafà R., Di Gregorio C., Fante R., Losi L. (1999). Microsatellite instability in multiple colorectal tumors. Int. J. Cancer.

[46] Keijzers G., Bakula D., Scheibye-Knudsen M. (2017). Monogenic Diseases of DNA Repair. N. Engl. J. Med..

[47] Nicolaides N.C., Littman S.J., Modrich P., Kinzler K.W., Vogelstein B. (1998). A naturally occurring hPMS2 mutation can confer a dominant negative mutator phenotype. Mol. Cell. Biol..

[48] Kadyrov F.A., Genschel J., Fang Y., Penland E., Edelmann W., Modrich P. (2009). A possible mechanism for exonuclease 1-independent eukaryotic mismatch repair. Proc. Natl. Acad. Sci. USA.

[49] Goellner E.M., Smith C.E., Campbell C.S., Hombauer H., Desai A., Putnam C.D., Kolodner R.D. (2014). PCNA and Msh2-Msh6 activate an Mlh1-Pms1 endonuclease pathway required for Exo1-independent mismatch repair. Mol. Cell.

[50] Saydam N., Kanagaraj R., Dietschy T., Garcia P.L., Peña-Diaz J., Shevelev I., Stagljar I., Janscak P. (2007). Physical and functional interactions between Werner syndrome helicase and mismatch-repair initiation factors. Nucleic Acids Res..

[51] Machwe A., Lozada E., Wold M.S., Li G.M., Orren D.K. (2011). Molecular cooperation between the Werner syndrome protein and replication protein A in relation to replication fork blockage. J. Biol. Chem..

[52] Kawasaki T., Ohnishi M., Suemoto Y., Kirkner G.J., Liu Z., Yamamoto H., Loda M., Fuchs C.S., Ogino S. (2008). WRN promoter methylation possibly connects mucinous differentiation, microsatellite instability and CpG island methylator phenotype in colorectal cancer. Mod. Pathol..

[53] Gray M.D., Shen J.C., Kamath-Loeb A.S., Blank A., Sopher B.L., Martin G.M., Oshima J., Loeb L.A. (1997). The Werner syndrome protein is a DNA helicase. Nat. Genet..

[54] Sommers J.A., Sharma S., Doherty K.M., Karmakar P., Yang Q., Kenny M.K., Harris C.C., Brosh R.M. (2005). p53 modulates RPA-dependent and RPA-independent WRN helicase activity. Cancer Res..

[55] Constantinou A., Tarsounas M., Karow J.K., Brosh R.M., Bohr V.A., Hickson I.D., West S.C. (2000). Werner’s syndrome protein (WRN) migrates Holliday junctions and co-localizes with RPA upon replication arrest. EMBO Rep..

[56] Cunniff C., Bassetti J.A., Ellis N.A. (2017). Bloom’s Syndrome: Clinical Spectrum, Molecular Pathogenesis, and Cancer Predisposition. Mol. Syndromol..

[57] Pedrazzi G., Perrera C., Blaser H., Kuster P., Marra G., Davies S.L., Ryu G.H., Freire R., Hickson I.D., Jiricny J. (2001). Direct association of Bloom’s syndrome gene product with the human mismatch repair protein MLH1. Nucleic Acids Res..

[58] Pedrazzi G., Bachrati C.Z., Selak N., Studer I., Petkovic M., Hickson I.D., Jiricny J., Stagljar I. (2003). The Bloom’s syndrome helicase interacts directly with the human DNA mismatch repair protein hMSH6. Biol. Chem..

[59] Sommers J.A., Banerjee T., Hinds T., Wan B., Wold M.S., Lei M., Brosh R.M. (2014). Novel function of the Fanconi anemia group J or RECQ1 helicase to disrupt protein-DNA complexes in a replication protein A-stimulated manner. J. Biol. Chem..

[60] Banerjee T., Sommers J.A., Huang J., Seidman M.M., Brosh R.M. (2015). Catalytic strand separation by RECQ1 is required for RPA-mediated response to replication stress. Curr. Biol..

[61] Doherty K.M., Sommers J.A., Gray M.D., Lee J.W., von Kobbe C., Thoma N.H., Kureekattil R.P., Kenny M.K., Brosh R.M. (2005). Physical and functional mapping of the replication protein a interaction domain of the werner and bloom syndrome helicases. J. Biol. Chem..

[62] Langland G., Kordich J., Creaney J., Goss K.H., Lillard-Wetherell K., Bebenek K., Kunkel T.A., Groden J. (2001). The Bloom’s syndrome protein (BLM) interacts with MLH1 but is not required for DNA mismatch repair. J. Biol. Chem..

[63] Bennett S.E., Umar A., Oshima J., Monnat R.J., Kunkel T.A. (1997). Mismatch repair in extracts of Werner syndrome cell lines. Cancer Res..

[64] Desai A., Gerson S. (2014). Exo1 independent DNA mismatch repair involves multiple compensatory nucleases. DNA Repair.

[65] Liu D., Frederiksen J.H., Liberti S.E., Lützen A., Keijzers G., Pena-Diaz J., Rasmussen L.J. (2017). Human DNA polymerase delta double-mutant D316A; E318A interferes with DNA mismatch repair in vitro. Nucleic Acids Res..

[66] Smith C.E., Mendillo M.L., Bowen N., Hombauer H., Campbell C.S., Desai A., Putnam C.D., Kolodner R.D. (2013). Dominant mutations in S. cerevisiae PMS1 identify the Mlh1- Pms1 endonuclease active site and an exonuclease 1-independent mismatch repair pathway. PLoS Genet..

[67] Tran P.T., Fey J.P., Erdeniz N., Gellon L., Boiteux S., Liskay R.M. (2007). A mutation in EXO1 defines separable roles in DNA mismatch repair and post-replication repair. DNA Repair.

[68] Karras G.I., Fumasoni M., Sienski G., Vanoli F., Branzei D., Jentsch S. (2013). Noncanonical role of the 9-1-1 clamp in the error-free DNA damage tolerance pathway. Mol. Cell.

[69] Friedberg E.C. (2001). How nucleotide excision repair protects against cancer. Nat. Rev. Cancer.

[70] Giannattasio M., Follonier C., Tourrière H., Puddu F., Lazzaro F., Pasero P., Lopes M., Plevani P., Muzi-Falconi M. (2010). Exo1 competes with repair synthesis, converts NER intermediates to long ssDNA gaps, and promotes checkpoint activation. Mol. Cell.

[71] Schaetzlein S., Chahwan R., Avdievich E., Roa S., Wei K., Eoff R.L., Sellers R.S., Clark A.B., Kunkel T.A., Scharff M.D. (2013). Mammalian Exo1 encodes both structural and catalytic functions that play distinct roles in essential biological processes. Proc. Natl. Acad. Sci. USA.

[72] Modrich P., Lahue R. (1996). Mismatch repair in replication fidelity, genetic recombination, and cancer biology. Annu. Rev. Biochem..

[73] El-Shemerly M., Hess D., Pyakurel A.K., Moselhy S., Ferrari S. (2008). ATR-dependent pathways control hEXO1 stability in response to stalled forks. Nucleic Acids Res..

[74] Mimitou E.P., Symington L.S. (2008). Sae2, Exo1 and Sgs1 collaborate in DNA double-strand break processing. Nature.

[75] Cheruiyot A., Paudyal S.C., Kim I.K., Sparks M., Ellenberger T., Piwnica-Worms H., You Z. (2015). Poly(ADP-ribose)-binding promotes Exo1 damage recruitment and suppresses its nuclease activities. DNA Repair.

[76] Zhang F., Shi J., Chen S.H., Bian C., Yu X. (2015). The PIN domain of EXO1 recognizes poly(ADP-ribose) in DNA damage response. Nucleic Acids Res..

[77] Liu Y., Kadyrov F.A., Modrich P. (2011). PARP-1 enhances the mismatch-dependence of 5’-directed excision in human mismatch repair in vitro. DNA Repair.

[78] Farah J.A., Cromie G.A., Smith G.R. (2009). Ctp1 and Exonuclease 1, alternative nucleases regulated by the MRN complex, are required for efficient meiotic recombination. Proc. Natl. Acad. Sci. USA.

[79] Nimonkar A.V., Genschel J., Kinoshita E., Polaczek P., Campbell J.L., Wyman C., Modrich P., Kowalczykowski S.C. (2011). BLM-DNA2-RPA-MRN and EXO1-BLM-RPA-MRN constitute two DNA end resection machineries for human DNA break repair. Genes Dev..

[80] Yang S.H., Zhou R., Campbell J., Chen J., Ha T., Paull T.T. (2013). The SOSS1 single-stranded DNA binding complex promotes DNA end resection in concert with Exo1. EMBO J..

[81] Williams B.R., Mirzoeva O.K., Morgan W.F., Lin J., Dunnick W., Petrini J.H. (2002). A murine model of Nijmegen breakage syndrome. Curr. Biol..

[82] Rein K., Yanez D.A., Terré B., Palenzuela L., Aivio S., Wei K., Edelmann W., Stark J.M., Stracker T.H. (2015). EXO1 is critical for embryogenesis and the DNA damage response in mice with a hypomorphic Nbs1 allele. Nucleic Acids Res..

[83] Wang Y., Putnam C.D., Kane M.F., Zhang W., Edelmann L., Russell R., Carrión D.V., Chin L., Kucherlapati R., Kolodner R.D. (2005). Mutation in Rpa1 results in defective DNA double-strand break repair, chromosomal instability and cancer in mice. Nat. Genet..

[84] Ferrari S. (2010). DNA end resection by CtIP and exonuclease 1 prevents genomic instability. EMBO Rep..

[85] Shim E.Y., Chung W.H., Nicolette M.L., Zhang Y., Davis M., Zhu Z., Paull T.T., Ira G., Lee S.E. (2010). Saccharomyces cerevisiae Mre11/Rad50/Xrs2 and Ku proteins regulate association of Exo1 and Dna2 with DNA breaks. EMBO J..

[86] Wang W., Daley J.M., Kwon Y., Krasner D.S., Sung P. (2017). Plasticity of the Mre11-Rad50-Xrs2-Sae2 nuclease ensemble in the processing of DNA-bound obstacles. Genes Dev..

[87] Rulten S.L., Grundy G.J. (2017). Non-homologous end joining: Common interaction sites and exchange of multiple factors in the DNA repair process. Bioessays.

[88] Kragelund B.B., Weterings E., Hartmann-Petersen R., Keijzers G. (2016). The Ku70/80 ring in Non-Homologous End-Joining: Easy to slip on, hard to remove. Front. Biosci..

[89] Shamanna R.A., Lu H., de Freitas J.K., Tian J., Croteau D.L., Bohr V.A. (2016). WRN regulates pathway choice between classical and alternative non-homologous end joining. Nat. Commun..

[90] Yu A.M., McVey M. (2010). Synthesis-dependent microhomology-mediated end joining accounts for multiple types of repair junctions. Nucleic Acids Res..

[91] Keijzers G., Maynard S., Shamanna R.A., Rasmussen L.J., Croteau D.L., Bohr V.A. (2014). The role of RecQ helicases in non-homologous end-joining. Crit. Rev. Biochem. Mol. Biol..

[92] Aggarwal M., Sommers J.A., Morris C., Brosh R.M. (2010). Delineation of WRN helicase function with EXO1 in the replicational stress response. DNA Repair.

[93] Iannascoli C., Palermo V., Murfuni I., Franchitto A., Pichierri P. (2015). The WRN exonuclease domain protects nascent strands from pathological MRE11/EXO1-dependent degradation. Nucleic Acids Res..

[94] Genschel J., Kadyrova L.Y., Iyer R.R., Dahal B.K., Kadyrov F.A., Modrich P. (2017). Interaction of proliferating cell nuclear antigen with PMS2 is required for MutLα activation and function in mismatch repair. Proc. Natl. Acad. Sci. USA.

[95] Chen X., Paudyal S.C., Chin R.I., You Z. (2013). PCNA promotes processive DNA end resection by Exo1. Nucleic Acids Res..

[96] Engels K., Giannattasio M., Muzi-Falconi M., Lopes M., Ferrari S. (2011). 14-3-3 Proteins regulate exonuclease 1-dependent processing of stalled replication forks. PLoS Genet..

[97] Andersen S.D., Keijzers G., Rampakakis E., Engels K., Luhn P., El-Shemerly M., Nielsen F.C., Du Y., May A., Bohr V.A. (2012). 14-3-3 checkpoint regulatory proteins interact specifically with DNA repair protein human exonuclease 1 (hEXO1) via a semi-conserved motif. DNA Repair.

[98] Chen X., Kim I.K., Honaker Y., Paudyal S.C., Koh W.K., Sparks M., Li S., Piwnica-Worms H., Ellenberger T., You Z. (2015). 14-3-3 proteins restrain the Exo1 nuclease to prevent overresection. J. Biol. Chem..

[99] Ngo G.H., Balakrishnan L., Dubarry M., Campbell J.L., Lydall D. (2014). The 9-1-1 checkpoint clamp stimulates DNA resection by Dna2-Sgs1 and Exo1. Nucleic Acids Res..

[100] Ngo G.H., Lydall D. (2015). The 9-1-1 checkpoint clamp coordinates resection at DNA double strand breaks. Nucleic Acids Res..

[101] Sun X., Zheng L., Shen B. (2002). Functional alterations of human exonuclease 1 mutants identified in atypical hereditary nonpolyposis colorectal cancer syndrome. Cancer Res..

[102] Hansen M.F., Johansen J., Bjørnevoll I., Sylvander A.E., Steinsbekk K.S., Sætrom P., Sandvik A.K., Drabløs F., Sjursen W. (2015). A novel POLE mutation associated with cancers of colon, pancreas, ovaries and small intestine. Fam. Cancer.

[103] Sokolenko A.P., Preobrazhenskaya E.V., Aleksakhina S.N., Iyevleva A.G., Mitiushkina N.V., Zaitseva O.A., Yatsuk O.S., Tiurin V.I., Strelkova T.N., Togo A.V. (2015). Candidate gene analysis of BRCA1/2 mutation-negative high-risk Russian breast cancer patients. Cancer Lett..

[104] Dong X., Li Y., Hess K.R., Abbruzzese J.L., Li D. (2011). DNA mismatch repair gene polymorphisms affect survival in pancreatic cancer. Oncologist.

[105] Yamamoto H., Hanafusa H., Ouchida M., Yano M., Suzuki H., Murakami M., Aoe M., Shimizu N., Nakachi K., Shimizu K. (2005). Single nucleotide polymorphisms in the EXO1 gene and risk of colorectal cancer in a Japanese population. Carcinogenesis.

[106] Bayram S., Akkız H., Bekar A., Akgöllü E., Yıldırım S. (2012). The significance of Exonuclease 1 K589E polymorphism on hepatocellular carcinoma susceptibility in the Turkish population: A case-control study. Mol. Biol. Rep..

[107] Tsai M.H., Tseng H.C., Liu C.S., Chang C.L., Tsai C.W., Tsou Y.A., Wang R.F., Lin C.C., Wang H.C., Chiu C.F. (2009). Interaction of Exo1 genotypes and smoking habit in oral cancer in Taiwan. Oral Oncol..

[108] Wang H.C., Chiu C.F., Tsai R.Y., Kuo Y.S., Chen H.S., Wang R.F., Tsai C.W., Chang C.H., Lin C.C., Bau D.T. (2009). Association of genetic polymorphisms of EXO1 gene with risk of breast cancer in Taiwan. Anticancer Res..

[109] Hsu N.Y., Wang H.C., Wang C.H., Chiu C.F., Tseng H.C., Liang S.Y., Tsai C.W., Lin C.C., Bau D.T. (2009). Lung cancer susceptibility and genetic polymorphisms of Exo1 gene in Taiwan. Anticancer Res..

[110] Jin G., Wang H., Hu Z., Liu H., Sun W., Ma H., Chen D., Miao R., Tian T., Jin L. (2008). Potentially functional polymorphisms of EXO1 and risk of lung cancer in a Chinese population: A case-control analysis. Lung Cancer.

[111] Bau D.T., Wang H.C., Liu C.S., Chang C.L., Chiang S.Y., Wang R.F., Tsai C.W., Lo Y.L., Hsiung C.A., Lin C.C. (2009). Single-nucleotide polymorphism of the Exo1 gene: Association with gastric cancer susceptibility and interaction with smoking in Taiwan. Chin. J. Physiol..

[112] Zhang M., Zhao D., Yan C., Zhang L., Liang C. (2016). Associations between Nine Polymorphisms in EXO1 and Cancer Susceptibility: A Systematic Review and Meta-Analysis of 39 Case-control Studies. Sci. Rep..

[113] Ibarrola-Villava M., Peña-Chilet M., Fernandez L.P., Aviles J.A., Mayor M., Martin-Gonzalez M., Gomez-Fernandez C., Casado B., Lazaro P., Lluch A. (2011). Genetic polymorphisms in DNA repair and oxidative stress pathways associated with malignant melanoma susceptibility. Eur. J. Cancer.

[114] Haghighi M.M., Taleghani M.Y., Mohebbi S.R., Vahedi M., Fatemi S.R., Zali N., Shemirani A.I., Zali M.R. (2010). Impact of EXO1 polymorphism in susceptibility to colorectal cancer. Genet. Test. Mol. Biomarkers.

[115] Alimirzaie S., Mohamadkhani A., Masoudi S., Sellars E., Boffetta P., Malekzadeh R., Akbari M.R., Pourshams A. (2018). Mutations in Known and Novel cancer Susceptibility Genes in Young Patients with Pancreatic Cancer. Arch. Iran Med..

[116] Michailidou K., Beesley J., Lindstrom S., Canisius S., Dennis J., Lush M.J., Maranian M.J., Bolla M.K., Wang Q., Shah M. (2015). Genome-wide association analysis of more than 120,000 individuals identifies 15 new susceptibility loci for breast cancer. Nat. Genet..

[117] Shi T., Jiang R., Wang P., Xu Y., Yin S., Cheng X., Zang R. (2017). Significant association of the EXO1 rs851797 polymorphism with clinical outcome of ovarian cancer. Onco. Targets Ther..

[118] Peltomäki P., Vasen H.F. (1997). Mutations predisposing to hereditary nonpolyposis colorectal cancer: Database and results of a collaborative study. The International Collaborative Group on Hereditary Nonpolyposis Colorectal Cancer. Gastroenterology.

[119] Shao H., Baitinger C., Soderblom E.J., Burdett V., Modrich P. (2014). Hydrolytic function of Exo1 in mammalian mismatch repair. Nucleic Acids Res..

[120] Bregenhorn S., Jiricny J. (2014). Biochemical characterization of a cancer-associated E109K missense variant of human exonuclease 1. Nucleic Acids Res..

[121] Axelsen J.B., Lotem J., Sachs L., Domany E. (2007). Genes overexpressed in different human solid cancers exhibit different tissue-specific expression profiles. Proc. Natl. Acad. Sci. USA.

[122] Dai Y., Tang Z., Yang Z., Zhang L., Deng Q., Zhang X., Yu Y., Liu X., Zhu J. (2018). EXO1 overexpression is associated with poor prognosis of hepatocellular carcinoma patients. Cell Cycle.

[123] Muthuswami M., Ramesh V., Banerjee S., Viveka Thangaraj S., Periasamy J., Bhaskar Rao D., Barnabas G.D., Raghavan S., Ganesan K. (2013). Breast tumors with elevated expression of 1q candidate genes confer poor clinical outcome and sensitivity to Ras/PI3K inhibition. PLoS ONE.

[124] de Sousa J.F., Torrieri R., Serafim R.B., Di Cristofaro L.F., Escanfella F.D., Ribeiro R., Zanette D.L., Paçó-Larson M.L., da Silva W.A., Tirapelli D.P. (2017). Expression of signautes of DNA repair genes correlate with survival prognosis of astrocytomapatients. Tumour. Biol..

[125] Yuan S.S., Hou M.F., Hsieh Y.C., Huang C.Y., Lee Y.C., Chen Y.J., Lo S. (2012). Role of Mre11 in cell proliferation, tumor invasion and DNA repair in breast cancer. J. Natl. Cancer Inst..

[126] Zhang K., Keymeulen S., Nelson R., Tong T.R., Yuan Y.C., Yun X., Liu Z., Lopez J., Raz D.J., Kim J.Y. (2018). Overexpression of Flap Endonuclease 1 Correlates with Enhanced Proliferation and Poor Prognosis of Non-Small-Cell Lung Cancer. Am. J. Pathol..

[127] Abdel-Fatah T.M., Russell R., Albarakati N., Maloney D.J., Dorjsuren D., Rueda O.M., Moseley P., Mohan V., Sun H., Abbotts R. (2014). Genomic and protein expression analysis reveals flap endonuclease 1 (FEN1) as a key biomarker in breast and ovarian cancer. Mol. Oncol..

